# Coping strategies among poverty-affected adolescents experiencing or at risk of depression and anxiety in Nepal: a qualitative study

**DOI:** 10.1186/s40359-025-03698-6

**Published:** 2025-12-01

**Authors:** Atuleisha Thapa, Bhola Teli, Ramya Pillutla, Rakesh Singh, Susma Baniya, Kreeti Budhathoki, Brandon Kohrt, Mark J.D. Jordans, Emily Garman, Crick Lund, Kamal Gautam, Sara Evans-Lacko, Nagendra P. Luitel

**Affiliations:** 1 Research Department, Transcultural Psychosocial Organization Nepal (TPO Nepal), Baluwatar, Kathmandu Nepal; 2 Centre for Mental Health Law and Policy, Indian Law Society, Pune, Pune India; 3 Department of Psychiatry and Behavioural Health, Centre for Global Mental Health Equity, George Washington University, Washington, D.C USA; 4 Health Service and Population Research Department, Centre for Global Mental Health, Institute of Psychiatry, Psychology and Neuroscience, King’s College London, London, UK; 5 Department of Psychiatry and Mental Health, Alan J Flisher Centre for Public Mental Health, University of Cape Town, Cape Town, South Africa; 6 Care Policy and Evaluation Centre, London School of Economics and Political Science, London, UK; 7 Department of Global Public Health, Karolinska Institutet, Stockholm, Sweden

**Keywords:** Adolescents, Depression and anxiety, Coping strategy, Nepal

## Abstract

**Introduction:**

Adolescents employ various coping strategies to manage stress, which can impact their short and long-term mental health. Economically disadvantaged adolescents are more prone to developing maladaptive coping mechanisms due to the persistent stressors associated with poverty. In Nepal, there is a lack of research on how adolescents experiencing or at risk of depression and anxiety cope with adverse situations. This study aims to explore how adolescents affected by poverty and at risk of experiencing mental health problems navigate difficulties within their homes, peer groups, and school environments.

**Methods:**

The study focused on adolescents aged 11 to 19 residing in informal squatter settlements in Kathmandu, Nepal. In-depth narrative interviews were conducted with 30 adolescents experiencing or at risk of depression and/or anxiety. In addition, journal entries documenting their experiences were collected weekly for 3–5 weeks post-interviews. Thematic and plot analysis were employed to identify the coping strategies utilised by adolescents in this context, which were then categorised according to coping theory.

**Results:**

The results indicated that adolescents employed a combination of adaptive and maladaptive coping strategies across different settings (home, school, and peer interactions), with variation in coping strategies used within each setting. *Problem solving* emerged as the most prevalent coping strategy across all settings. Adolescents tended to exhibit more *submission* and *helplessness* at home, more *isolation* and *opposition* among peers, and more *accommodation* and *opposition* at school. Adolescents aged 14–16 displayed more maladaptive coping strategies compared to younger (11 to 13 years) and older adolescents (17 to 19 years). While girls and boys utilised similar adaptive and maladaptive coping strategies, girls leaned more towards *avoidance-escape* and *helplessness*, whereas boys favoured *opposition* and *isolation*.

**Conclusion:**

The results highlight the range of adaptive and maladaptive coping strategies used by adolescents to manage stress in circumstances of urban poverty in Nepal. The study recommends focused psychological interventions which equip adolescents with adaptive coping strategies and stress appraisal techniques that help them manage difficulties effectively.

**Supplementary Information:**

The online version contains supplementary material available at 10.1186/s40359-025-03698-6.

## Introduction

Adolescence is a crucial phase of life marked by rapid physical, cognitive, emotional, and psychosocial development. During this period, adolescents are more susceptible to stress, increasing their risk of mental health issues like depression and anxiety. Globally, 14% of adolescents aged 10–19 experience a mental disorder, with estimated point prevalence rates of depression and anxiety disorders at 2.4% and 4.9% respectively [1]. If left unaddressed, these conditions can hinder adolescents from leading fulfilling lives and may even lead to suicide, which is the third leading cause of death among youth aged 15–29 [2]. Therefore, it is essential for adolescents to learn effective coping strategies to prevent and manage depression and anxiety. Around 90% of the world’s 1.2 billion adolescents, aged 10–19, live in low- and middle-income countries (LMICs) [3]. Recent research has shown a bidirectional relationship between mental illness and poverty [4, 5], with poverty being a significant risk factor for depression and anxiety [5]. Poverty is more prevalent in LMICs [6], adding to the risk of depression and anxiety among adolescents living in these countries [7, 8]. Nepal has the 4th highest suicide rate in the Southeast Asia Region [9], with 5.2% of adolescents aged 13–17 experiencing mental disorders [10]. However, there is a lack of research on how poverty-affected adolescents in Nepal cope with depression and/or anxiety.

Lazarus and Folkman [11] defined coping as the thoughts and actions a person uses to deal with stress. It involves managing problems or emotions that feel challenging or overwhelming. There is no “right” or “wrong” way to cope with adversity, but coping strategies involving self-blame, isolation, or aggressive behavior are generally considered maladaptive. Prolonged use of maladaptive coping strategies such as helplessness, rumination, and social withdrawal can have negative effects on adolescents’ physical and mental well-being. For example, avoidance is linked to a higher prevalence of depressive symptoms and poor adaptation [12, 13]. On the other hand, coping strategies involving constructive actions or positive reappraisal are considered adaptive [14]. Adaptive coping strategies such as problem solving and seeking support can promote increased stress resistance, self-reliance, and perceived control [14] and are associated with fewer depressive symptoms in adolescents [13]. Previous research suggests that economically disadvantaged adolescents tend to rely more on avoidance and withdrawal strategies when dealing with stress, which can have negative effects on their mental health in the long term [15, 16]. This tendency arises from continued exposure to multiple stressors associated with poverty, such as family conflict, harsh parenting, and maternal depression, which can diminish the development of self-regulation skills and coping capacities [15, 16]. As a result, maladaptive coping behaviours become entrenched and are likely to persist over time. Interventions are needed to alter these patterns and promote more adaptive coping strategies [17].

This study was conducted as part of the ALIVE (Improving Adolescent mentaL health by reducing the Impact of poverty) project [18], which aims to develop and pilot-test an intervention that equips adolescents with skills to escape poverty and strengthens self-regulation, thereby preventing adolescent depression and anxiety in urban settings in Colombia, Nepal, and South Africa. The study also aims to address a gap in the literature by exploring the experiences of adolescents affected by poverty in Nepal who are dealing with or at risk of depression and/or anxiety. By understanding the prevalence and nature of coping strategies adolescents employ in their daily lives, the study seeks to fill a gap in the literature and inform interventions to promote mental well-being in this population.

## Methods

### Setting

The study was conducted in urban poverty settings, specifically in squatter settlements in Kathmandu Valley, including Kapan, Teku, Balkhu, Balaju, Manohara, and Sinamangal. Squatter settlements are informal communities located alongside riverbanks and their tributaries in Kathmandu Valley, where residents live without legal rights. The prevalence of multidimensional poverty in these areas is very high due to poor housing quality, overcrowding, and inadequate access to safe water, sanitation, and other infrastructure [19].

### Study design

This study utilized narrative interviewing to collect in-depth personal accounts from adolescents regarding their coping strategies. Reflective journaling and iterative short interviews were also employed to improve the longitudinal understanding of their experiences. In these interviews, research participants were asked to briefly describe what they did, thought, and felt during the given timeframe.

### Participants and recruitment

The study involved adolescents aged 11–19 years who were either experiencing or at risk of depression and/or anxiety, residing in selected squatter settlements of the Kathmandu Valley, such as Teku (*n* = 5), Balkhu (*n* = 5), Kapan (*n* = 6), Manohara (*n* = 6), Sinamangal (*n* = 5), and Balaju (*n* = 3). Research assistants visited the selected settlements, purposively selected households, and assessed the eligibility of adolescents within those households. If an eligible adolescent was identified, they provided detailed information about the study and obtained informed consent from both the adolescents and their caregivers if the participants were under 18 years old. The Patient Health Questionnaire for Adolescents (PHQ-A) and the Generalized Anxiety Disorder Scale (GAD-7) were used to assess symptoms of depression and anxiety. Adolescents with a PHQ-A score of 15 or higher were considered to have depression, while those with a GAD-7 score of 9 or higher were considered to have anxiety, based on validated cut-off scores for Nepali adolescents [20]. Participants scoring between 6–14 on the PHQ-A and 4–8 on the GAD-7 were considered at risk for depression or anxiety symptoms. In cases where participants reported suicidal thoughts, self-harm, suicide attempts, or experiences of abuse, researchers followed a safety protocol to assess risk and provide necessary psychological support. All interviews were conducted in a private and confidential space within the household.

### Interview guide

We developed a semi-structured interview guide based on the framework by Conover and Daiute [20] to explore adolescents’ narratives of challenging situations in their daily lives across three settings: home, school, and with peers. Our goal was to capture experiences beyond interpersonal conflicts, including strategies for achieving their goals, both positive and negative to gain a comprehensive understanding of how adolescents cope with life challenges. We used concise prompts tailored to adolescents’ experiences to elicit narratives, following the approach outlined by Conover and Daiute [20] (e.g., “Describe a time when you felt upset with your caregivers”). These prompts were followed by questions about the challenges faced, reactions, outcomes, and ideal responses. Additionally, we also included follow-up questions to gather reflections on any changes or challenges encountered post-interview. The guide was pilot-tested for cultural sensitivity and clarity for participants with varying education levels. The interview guide is attached as a supplement file.

### Interview and journal activity procedures

After recruiting participants based on their PHQ-A and GAD-7 scores, trained Research Assitants conducted in-depth narrative interviews in participants’ homes using the provided interview guides. Weekly follow-up visits were then carried out to collect journal entries or conduct additional interviews for up to five weeks to gather more detailed information about participants’ ongoing difficulties and stressors. Participants were given the option to complete their journal entries independently in a diary or share their experiences orally with RAs on a weekly basis. With the exception of one participant who withdrew after the initial interview, each of the remaining 29 participants completed between two and five follow-up visits, resulting in a total of 97 visits. There were 91 oral updates and only 6 journal entries, as many participants either lost their journal notebooks or found it challenging to express their experiences in writing, preferring short interviews instead. All narrative and follow-up interviews were audio-recorded, with narrative interviews lasting 45–90 minutes and follow-up interviews lasting 5–30 minutes. Researchers reviewed audio recordings periodically and concluded interviews once thematic saturation was achieved. Additionally, socio-demographic information such as age, gender, caste, ethnicity, religion, address, school type, grade, parents’ occupations, and parents’ education levels were collected from all participants following the initial interview.

### Data analysis

After each interview, the Research Assistants transcribed the audio recordings verbatim in Nepali. These transcripts were then translated into English by separate translators. The research team reviewed the translations to ensure that the English version accurately reflected the original meaning in Nepali. To preserve the essence of Nepali idioms and expressions, translators occasionally included both the English translation and the corresponding Romanized Nepali terms in brackets. The final English transcripts were entered into NVivo software for plot analysis and thematic analysis.Plot analysis is used to examine the structure and development of adolescents’ narratives, focusing on key elements such as initiating actions, complicating events, and resolution strategies [20]. Plot analysis allows a deeper exploration of coping trajectories and patterns that may not be apparent through thematic analysis alone, enhancing our understanding of how adolescents navigate challenges within their social and environmental contexts.

Before conducting the analysis, we adapted Conover and Daiute’s definitions of plot elements [20] to better suit our data and extract information on the coping process demonstrated by participants. We focused on the stressors faced by the participants (*initiating action*, *complicating action*), their immediate psychological reaction when they first encountered the stressor (*desired strategy – “I wanted to hit the teacher”*), their actual attempt at coping with the stressor (*actual strategy – “I argued with the teacher”*), and the response they deemed ideal upon reflection (*ideal strategy – “Think positively about what the teacher said”*). Three researchers (AT, BT, RP) coded five transcripts to refine definitions and specify inclusion criteria for plot elements. After achieving 85% agreement on inter-coder reliability (ICR), we divided the remaining transcripts and identified narratives within each interview and coded for these plot elements within each narrative. We created “meaning units” [21] from the plot codes for ‘Stressor’ and ‘Strategy’ and entered them into an Excel spreadsheet. We then classified strategies reported by participants into the 12 “coping families” based on Skinner and Zimmer-Gembeck’s coping theory [22], consolidating the data in the same master spreadsheet. Among them, *accommodation*, *information seeking*, *negotiation*, *problem solving*, *self-comforting*, and *support seeking* are considered adaptive, while *avoidance-escape*, *delegation*, *helplessness*, *social isolation*, *opposition*, and *submission* are considered maladaptive. 

Subsequently, we quantitatively assessed the frequency of *desired*, *actual*, and *ideal* strategies reported across all settings, comparing the prevalence of adaptive versus maladaptive coping strategies and the most frequently used coping strategy families in each setting (i.e., home, peer, school settings). Additionally, we quantitatively analyzed gender and age differences in *actual* coping strategies. Integrating quantitative summaries into qualitative results helps identify recurring patterns and deviations that may not be obvious from narrative data alone. This approach improves the transparency and credibility of the findings by reducing potential bias and providing empirical support for interpretations. Quantification also helps focus on key themes, allows for cautious generalization within the study context, and presents results clearly and visually [23, 24].

For thematic analysis, the same three researchers (AT, BT, RP) analyzed the same five transcripts to identify emergent codes. After discussing and selecting the emergent codes, an initial codebook was developed. Subsequently, three transcripts were analyzed using deductive and inductive methods, and the codes were compared, disagreements resolved, and the codebook revised. This iterative process continued with additional transcripts until ICR was established based on three transcripts (10% of the sample), with final Cohen Kappa coefficients ranging between 0.63 and 0.73. The remaining transcripts were then coded using the finalized codebook. A final codebook was developed by merging, removing, and renaming codes as necessary, and organizing codes under parent codes. The final codebook consists of 23 parent codes, 129 child codes, descriptions, example quotes, and exclusion criteria. 

## Results

Table 1 presents the socio-demographic and mental health characteristics of the participants. More than half (56.7%) were girls and 60% belonged to the Janajati caste/ethnic group. A minority were at risk for depression and anxiety (26.7%) and had anxiety (26.7%).

**Table 1:** Socio-demographic characteristics of the participants

	*N* (30)	%
*Gender*		
Boy	13	43.3
Girl	17	56.7
*Age group*		
11–13	10	33.3
14–16	11	36.7
17–19	9	30.0
*Caste/ethnicity*		
Brahman/Chhetri	6	20.0
Janajati (Limbu, Shrestha, Magar, Tamang, Lama, Rai)	18	60.0
Dalit/Madhesi/Muslim (Ram, Sunar, Nepali, Mahato, Khatun)	6	20.0
*Completed education/grades*		
Five	3	10.0
Six	4	13.3
Seven	7	23.3
Eight	4	13.3
Nine	4	13.3
Ten or higher	8	26.7
*Status of depression and/or anxiety*		
At risk for depression	7	23.3
At risk for anxiety	4	13.3
A risk for both depression and anxiety	8	26.7
Lived experience with depression	2	6.7
Lived experience with anxiety	8	26.7
Lived experience with both depression and anxiety	1	3.3

### Results from thematic analysis

The results indicate that adolescents used a variety of coping strategies within each of the 12 coping families outlined by Skinner et al. [25]. Table 2 shows that at least four different coping strategy codes were identified for *opposition*, *submission*, and *problem solving*, while only one coping strategy code was identified for *avoidance-escape*, *delegation*, and *self-comforting*. Additionally, three non-strategy codes captured participants’ reports on the impact of using problem solving and support-seeking.

**Table 2:** Coping strategies identified thematically

Coping family (14, 24)	Coping strategies identified from narratives
Adaptive Strategies	
Accommodation (adjusting personal preferences to match situational constraints; acceptance; cognitive restructuring; distraction)	Positively responding to negative situations (*n* = 23, 76.7%), Distracting self (*n* = 14, 46.7%), Coming to terms with the situation (*n* = 4, 13.3%)
Information seeking (learning more about a stressful condition or situation as well as strategies for intervention; observation; monitoring)	Understanding situation before reacting (*n* = 4, 13.3%), Thinking rationally to decide (*n* = 6, 20%)
Negotiation (working out a compromise between one’s priorities and situational constraints; priority setting; taking other’s perspective)	Tolerating or not worrying (*n* = 18, 60%), Trying to avoid punishment (*n* = 8, 26.7%), Yielding (*n* = 3, 10%)
Problem solving (making efforts or taking actions directed at instrumentally changing the stressful situation; planning; strategizing)	Talking with adult (*n* = 18, 60%), Talking with peers (*n* = 17, 56.6%), Taking a stand (*n* = 19, 63.3%), Complaining to higher authority (*n* = 8, 26.7%), Talking not helpful^a^ (*n* = 19, 63.3%)
Self-comforting (expressing and regulating emotions constructively to alleviate emotional distress; self-soothing; positive self-talk)	Self-calming activities (*n* = 8, 26.7%)
Support seeking (actively attempting to receive instrumental help, advice, comfort or contact from parents, peers, spouses, professionals, and God)	Seeking support from family (*n* = 17, 56.6%), seeking support from peers (*n* = 24, 80%), seeking support from adults (*n* = 9, 30%), Elders not supporting adolescents^a^ (*n* = 8, 26.7%), Unsupportive peers^a^ (*n* = 4, 13.3%)
Maladaptive Strategies	
Avoidance-escape (making efforts to disengage or stay away from the stressful transaction; cognitive avoidance; denial; wishful thinking)	Leaving the situation temporarily (*n* = 20, 66.7%)
Delegation (over-reliance on others to cope with stress while focusing on the distressing aspects of the situation; dependence; self-pity; complaining; whining)	A few quotes (*n* = 4) from Brooding about a situation (*n* = 21, 70%)
Helplessness (relinquishing control or withdrawing active attempts to change the situation; confusion; passivity; cognitive exhaustion)	Staying quiet and not reacting (*n* = 17, 56.6%), Not taking any action (*n* = 17, 56.6%), Feeling helpless (*n* = 7, 23.3%)
Opposition (actions focused on attacking or combating the perceived source of the stress; aggression; projection; revenge; defiance; venting)	Shouting or beating (*n* = 20, 66.7%), Expressing dislike towards adults (*n* = 11, 36.7%), Expressing frustration (*n* = 9, 30%), Backbiting about adults (*n* = 6, 20%), Retaliating (*n* = 5, 16.7%), Projecting anger on others (*n* = 2, 6.7%),
Social isolation (withdrawing from other people or preventing them from knowing about a stressful situation; avoiding others; concealment)	Avoiding interaction (*n* = 26, 86.7%), Social withdrawal (*n* = 15, 50%)
Submission (grudgingly surrendering to stressful events with a passive, repetitive focus on the negative, damaging aspects; negative thinking; self-blame)	Wanting to leave the situation permanently (*n* = 22, 73.3%), Brooding about a situation (*n* = 21, 70%), Regretting their actions (*n* = 9, 30%), Wishing they were not born (*n* = 5, 16.7%), Blaming themselves (*n* = 4, 13.3%)

^a^Non-strategy codes capturing participants’ reports on the impact of using problem solving and support-seeking

### Adaptive strategies

Participants’ use of *accommodation* included responding positively to negative situations (cognitive restructuring), coming to terms with the situation (acceptance), and distraction.

*We may get angry at our family*,* but what if they die tomorrow? So*,* we should love and respect them now. – 15-year-old girl*.

*When I sense that I am going to be reminded of my problems*,* I immediately listen to music or go outside to play or learn the guitar. – 16-year-old boy*.

Instances of *information seeking* involved engaging in rational thought and taking the means to understand the situation before reacting to the problem.

*If our friend does something bad to us*,* first*,* we should figure out who is at fault. We shouldn’t fight with friends without understanding the situation. – 16-year-old boy*.

On the other hand, *negotiation* involved participants enduring, ignoring, or tolerating a stressful situation, or choosing to not worry about it. It also included yielding, where adolescents gave in to the situation or the person when they did not necessarily want to.

*My older sister treated me harshly and… said*,* “It would not affect me if you left [the family] because you are nothing to me. You don’t exist to me”. After that*,* I quietly did the chores without talking to anyone. But [later] my sister came to speak to me first*,* and I talked [to her] because I didn’t want to show an attitude to a family member. – 13-year-old girl*.

*Problem solving* strategies included taking instrumental action (such as complaining to higher authority) and direct communication, such as talking to or confronting the individual causing stress.

*At first*,* I felt hurt and wanted to cry*,* but later I realized he is a friend and friends do this type of thing*,* so I went and talked to him. – 17-year-old girl*.

However, participants noted that these strategies did not always lead to a resolution of the problem.

*As I was the only one in the house at that time*,* my aunt thought that I had stolen her accessory. But even when I told her I did not steal it*,* she did not believe me. – 12-year-old girl*.

Participants’ use of *self-comforting* involved calming activities like deep breathing and playing musical instruments.

*(After having a fight with my friends) I keep quiet*,* close my eyes*,* and sit on the bench. I take deep breaths with my eyes closed and I don’t respond even if anyone calls me. I do this for 10 minutes. – 12-year-old girl*.

Instances of *support-seeking* involved asking for advice and sharing their stressors with peers, family members, teachers, and neighbours, which helped many adolescents cope with their problems and feel happy and supported. However, when they did not receive the expected support from their peers and elders, adolescents reported feeling sad, upset, angry, and hurt.

*My friend made fun of me while I was sharing something sad about my personal life. This is the only reason I was angry with my friend this week. – 13-year-old boy*.

### Maladaptive strategies

*Avoidance-escape* included behaviours such as walking or running away from the classroom or home, sleeping over at a friend’s place, or roaming around the neighbourhood, in order to physically avoid or separate oneself from the place associated with the stressor.

*Sometimes*,* when my mother scolds me excessively*,* I think of running away from home… I don’t remember the particular day that she scolded me*,* but I felt like running away from my house*,* so I stayed outside.* – 12-year-old girl.

Interestingly, in contrast to the previous example, one participant associated the act of roaming around with the cognitive—rather than physical—avoidance of the stressor, as illustrated in the quote below.

*He (referring to a friend) might feel like he doesn’t want to stay at home and just leave and roam around because his teacher physically punished him.* – 13-year-old boy.

Only a few instances of *delegation were identified among instances of submission within the same code “Brooding about a situation”*, where adolescents surrendered to self-pity (“why me?”).

*The night that I got slapped by my sister*,* I thought so much about why she hit me in front of everyone during the festival and why I was so unlucky. I was constantly thinking about it until I fell asleep. – 15-year-old girl*.

*Helplessness* was characterised by feelings and perceptions of a lack of agency in a stressful situation as well as participants staying quiet and not reacting in response to aggression by others.

*My classmates used to humiliate me because I used to study in a lower grade despite looking big (i.e.*,* older)*,* but I didn’t say anything even though I was angry. They used to yell at me and hit me*,* but I stayed quiet. – 16-year-old boy*.

On the other hand, *opposition* included aggressive strategies like shouting, hitting, and expressing frustration.

*Two of my friends came to where I was standing and started laughing and shouting at me. I was angry*,* and my hands and body were shaking. I couldn’t control myself*,* and without realizing it*,* I hit them first.* – 17-year-old girl.

When adolescents were scolded or punished by adults, typically teachers, some would respond by speaking rudely, gossiping about them, skipping school as an act of rebellion, and avoiding specific teachers’ classes to show their dislike.

*They (referring to her classmates) never used to complete their homework… They used to tear the book of different subjects taught by the teacher with whom they were angry.* – 13-year-old girl.

*But among friends*,* we used to call the principal “budhi” (old lady) and backbite her*. – 14-year-old girl.

*Social isolation* involved participants avoiding any interaction with the person who caused them stress, such as not talking to them and refusing to listen to any requests made by them. It also included withdrawing from social interactions altogether, such as staying alone in a classroom, shutting oneself in one’s room all day, or expressing the desire to not be around people at all.

*She (her classmate) sits by herself*,* avoids interacting with the person who injured her*,* ceases to laugh*,* and even ceases to converse with other people in the same manner as before.* – 13-year-old girl.

*Submission* was characterized by participants brooding about or ruminating over the situation, expressing the desire to leave the situation permanently due to the emotional toll it has on them, as well as feeling deep regret or guilt for their actions which they believed were wrong or harmful.

*I missed my [deceased] mother and cried and wished I was never born because I was so unlucky.* – 15-year-old girl.

…*she always got scolded by her mother for no reason*,* and she felt like running away from her house and living far from her family.* – 12-year-old girl.

### Results from plot analysis

We identified a total of 252 narratives, 44.4% of which were in the home setting, 35.3% in the Peer setting, and 20.2% in the school setting, as demonstrated in Table 3. The proportion of narratives reported in each setting by early adolescents (11–13 years old), middle adolescents (14–16 years old), and older adolescents (17–19 years old) consistently followed the pattern: Home > Peer > School, with girls following the same pattern and boys following a Peer > Home > School pattern.

**Table 3:** Proportion of narratives reported by adolescents across settings by age group and gender

	Home	Peer	School
Overall	44.4%	35.3%	20.2%
*Age group*			
Early adolescents	43.2%	33.3%	23.5%
Middle adolescents	45.0%	36.7%	18.3%
Older adolescents	45.2%	35.5%	19.4%
*Gender*			
Female	50.0%	30.0%	20.0%
Male	34.8%	44.6%	20.7%

**Fig. 1:**
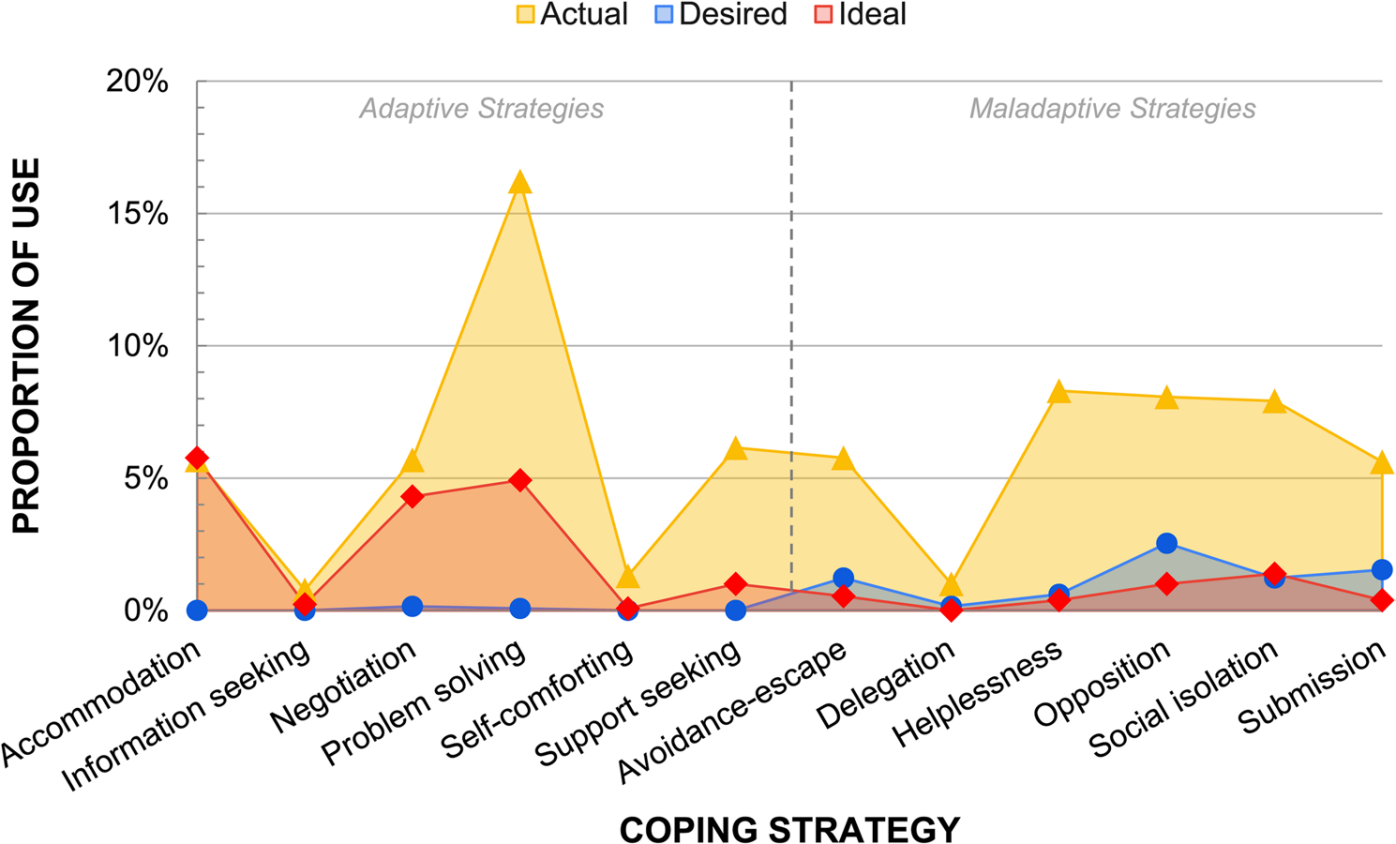
Comparison of reported *desired*, *actual*, and *ideal *coping strategies across all settings

A total of 943 strategies were identified. *Actual* strategies were mentioned 72.5% of the time, followed by *ideal* strategies at 20% and *desired* strategies at 7.5%. Figure 1 illustrates the differences in the proportion of adaptive and maladaptive coping strategies across different types. The *desired* strategies, which reflected participants’ psychological state when facing the stressor, were predominantly maladaptive (96.9%). In contrast, the *ideal* strategies reported by participants were mostly adaptive (81.5%), with a small portion being maladaptive (18.5%). Finally, the *actual* strategies reported were almost evenly split between adaptive and maladaptive coping strategies.

### Use of reported actual coping strategies

**Fig. 2:**
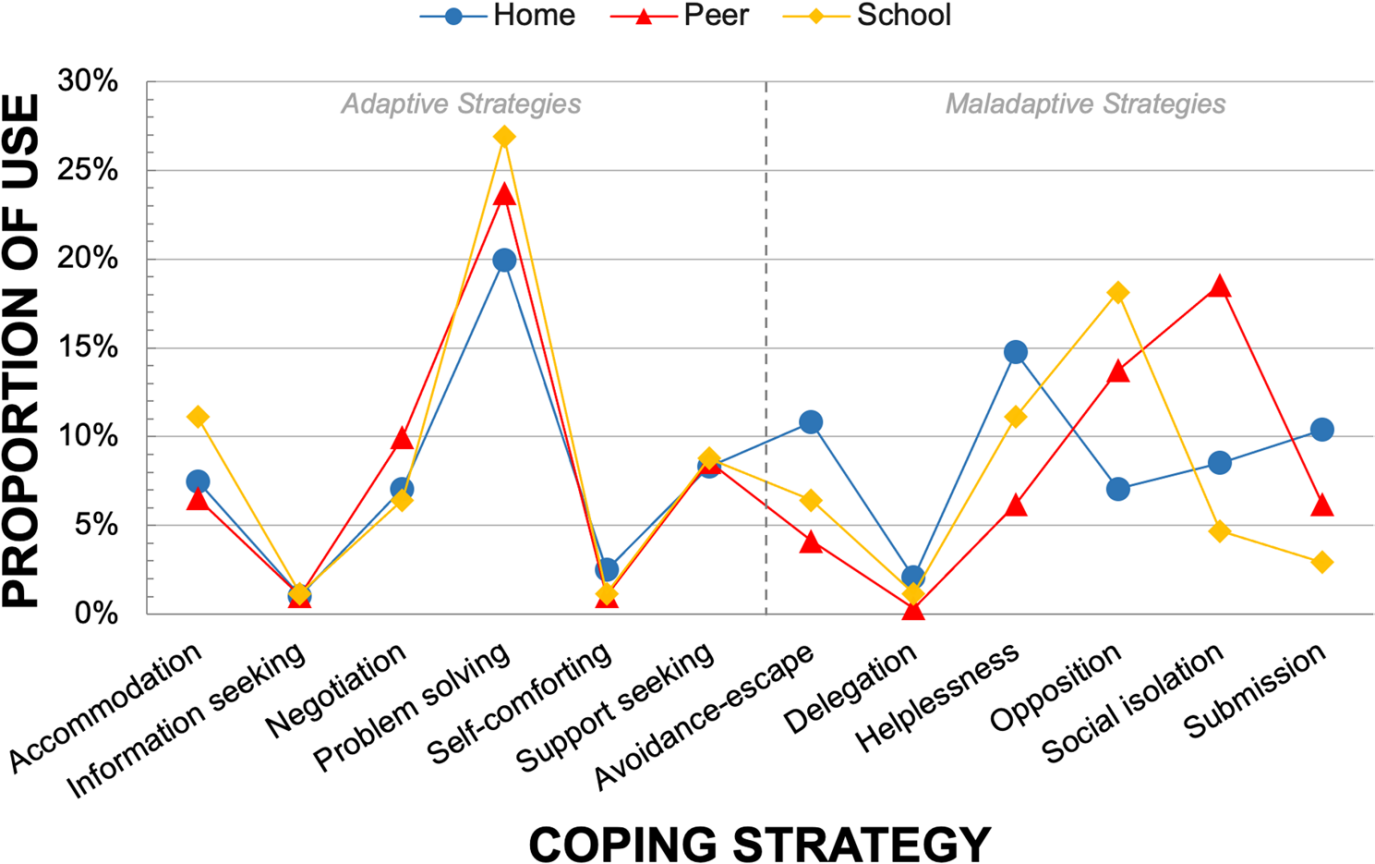
Comparison of reported *actual *coping strategies across Home, School and Peer contexts

Participants used a similar amount of maladaptive and adaptive coping strategies across all settings, with 49.4% adaptive and 50.6% maladaptive strategies. *Problem solving* was the most common coping strategy in all settings, accounting for over 20% of strategies used, whereas the least common strategies were *information seeking*, *self-comforting*, and *delegation*. However, the use of other coping strategies varied within settings, as seen in Fig. 2.

At Home, participants used more maladaptive coping strategies (53.6%) than adaptive ones (46.4%), including *helplessness*, *submission*, *avoidance-escape*, and *social isolation*. Besides *problem solving*, they also employed other adaptive strategies like *support seeking* and *accommodation*. In the Peer setting, there was an almost equal distribution of adaptive (50.9%) and maladaptive (49.1%) coping strategies. *Social isolation* and *opposition* were the most common maladaptive strategies, while *negotiation* and *support seeking* were the most common adaptive strategies after *problem solving*. In the School setting, participants mainly used adaptive coping strategies (55.6%) over maladaptive ones (44.4%). *Accommodation* and *support seeking* were the most common adaptive strategies after *problem solving*, while *opposition* and *helplessness* were the most common maladaptive strategies.

### Coping strategies by age

**Fig. 3:**
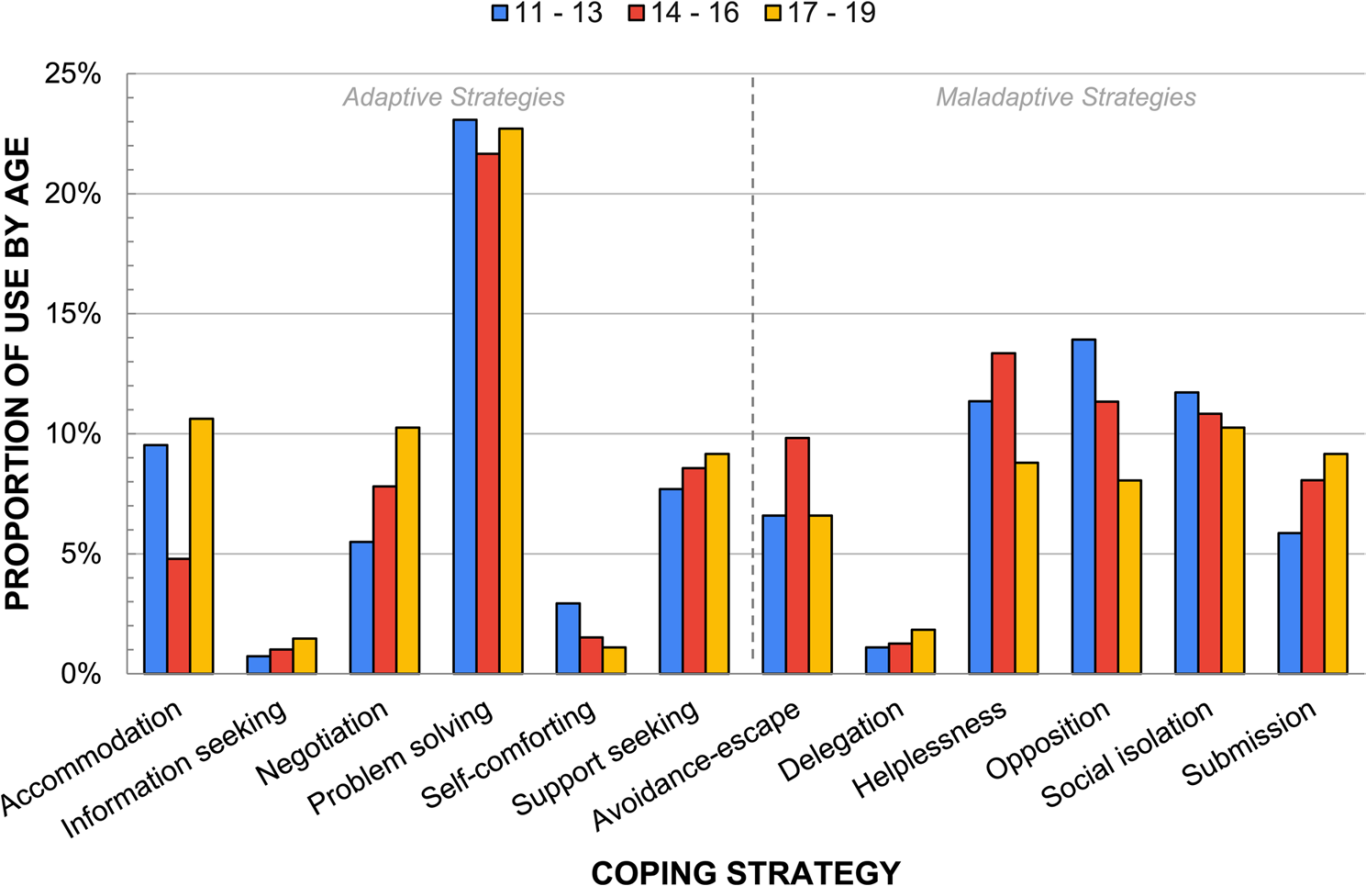
Comparison of reported *actual* coping strategies by age

As shown in Table 3, all age groups reported similar proportions of narratives in Home (43.2%–45.2%), Peer (33.3%–36.7%), and School (18.3%–23.5%) settings. The differences in reported *actual* coping strategies by age are depicted in Fig. 3. Early adolescents utilized adaptive coping (50.5%) almost as often as maladaptive coping (49.5%). This age group employed *problem solving* and *self-comforting* as adaptive coping strategies, and *opposition* and *social isolation* as maladaptive coping strategies slightly more than older age groups. In contrast, middle adolescents used relatively more maladaptive coping (54.7%) than adaptive coping (45.3%). They relied on *helplessness* and *avoidance-escape* more frequently and *accommodation* less, compared to other age groups. Finally, older adolescents employed more adaptive coping (55.3%) than maladaptive coping (44.7%). They utilized *accommodation*, *negotiation*, *support-seeking*, and *information-seeking* more frequently than younger age groups. They also used *submission* and *delegation* more than younger adolescents.

### Coping strategies by gender

**Fig. 4:**
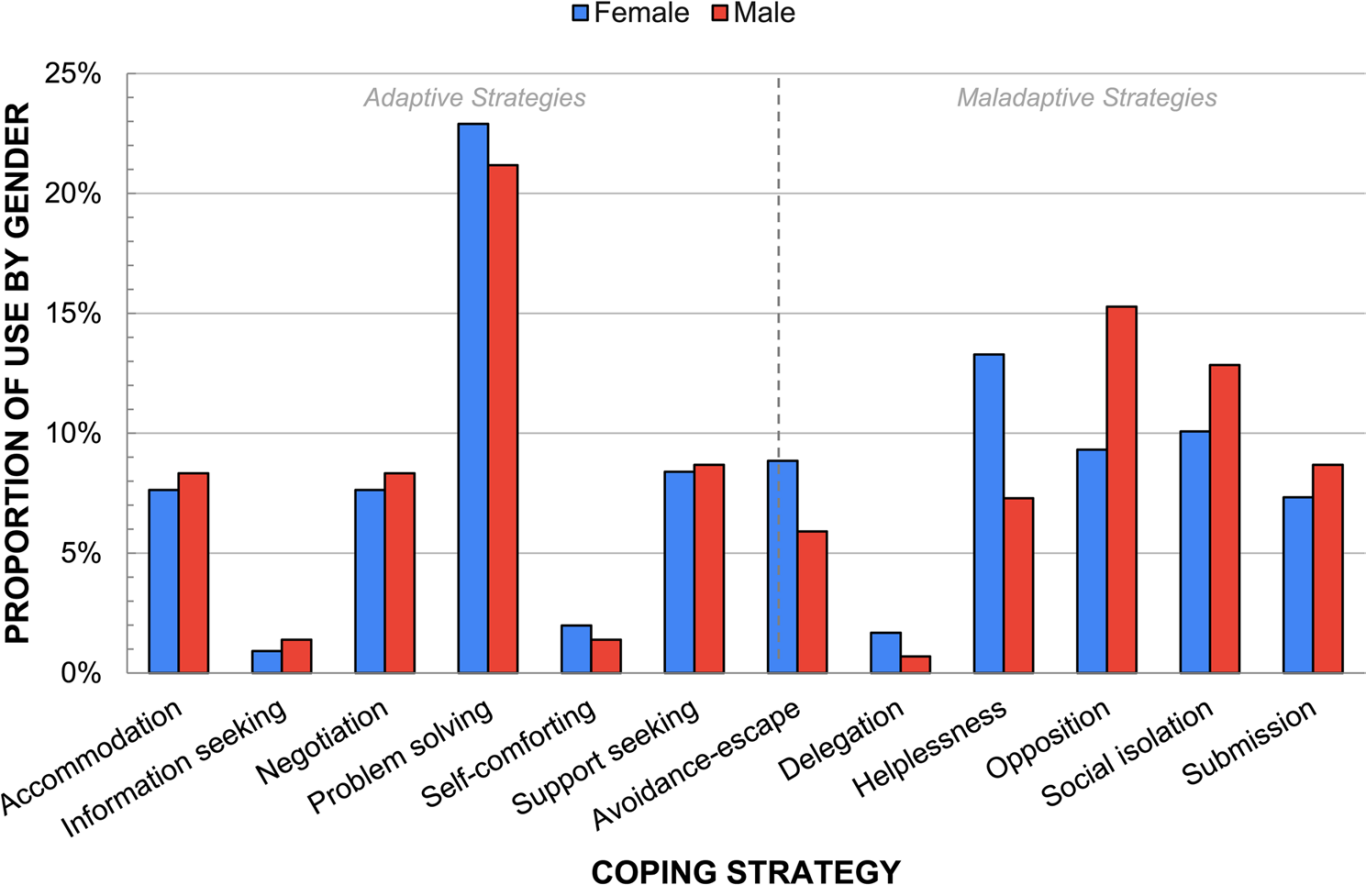
Comparison of reported *actual* coping strategies by gender

As shown in Table 3, girls reported a higher proportion of home narratives (50.0%) compared to Peer (30.0%), while boys reported more Peer narratives (44.6%) than Home (34.8%). Both genders reported a similar proportion of School narratives. Figure 4 shows the differences in reported actual coping strategies by gender. Boys utilized adaptive coping strategies (49.5%) almost as frequently as girls (49.3%), with girls using *problem solving* slightly more than boys. Girls tended to rely more on maladaptive coping strategies such as *helplessness*, *avoidance-escape*, and *delegation*, whereas boys exhibited higher usage of *opposition*, *social isolation* and *submission* compared to girls.

## Discussion

This study is the first to explore coping strategies among Nepalese adolescents living in circumstances of poverty and experiencing or at risk of depression and anxiety. Using thematic analysis, we identified distinct coping strategies aligned with Skinner et al.'s [25] 12 coping categories. Adolescents reported using a mix of adaptive and maladaptive coping strategies, with *problem solving* being the most common strategy in Home, Peer, and School settings, and *self-comforting*, *information seeking*, and *delegation* being the least common. Gender and age were not associated with coping strategies, but 14–16-year-old adolescents tended to use slightly more maladaptive strategies than their younger or older peers. We observed a distinction between *desired* and *ideal* coping strategies, with *desired* strategies leaning towards maladaptive (e.g., *opposition*, *submission*) and *ideal* strategies towards adaptive ones (e.g., *accommodation*, *problem solving*).

The presence of *desired*, *actual*, and *ideal* coping strategies in participants’ narratives indicates that coping is a dynamic process [26, 27]. Adolescents initially exhibited a reflex-like psychological response to stressors before attempting to adapt their response and subsequently engage in a reappraisal to determine what would have been an ideal response. The predominance of maladaptive *desired* strategies suggests that coping responses are influenced by strong emotions aligning with the findings of Conover and Daiute [20]. Conversely, the prevalence of adaptive *ideal* strategies indicates that participants possess knowledge of various coping strategies and can identify more effective ways of coping when removed from the stressful situation. This is consistent with Conover and Daiute's [20] observation that adolescents reported resolution-oriented strategies when taking on an ‘advisor’ role in a narrative. The findings on *ideal* strategies underscore that effective coping goes beyond knowing and implementing strategies but also recognizing that adaptive methods may not always be feasible in certain contexts. Furthermore, the equal distribution of adaptive and maladaptive *actual* coping strategies demonstrates that even in the moment, adolescents can evaluate and adjust their responses opting for more adaptive strategies.

Building on previous research [28], our study indicates that adolescents use various coping strategies to manage stress, emphasizing the importance of a flexible coping repertoire in enhancing adolescent resilience to complex stressors. While having access to a range of coping strategies should help adolescents overcome challenges [27], our findings suggest that relying on maladaptive coping strategies, even at a similar frequency to adaptive coping, may indicate a reduced sense of control or agency when faced with stressors. This highlights the contextual nature of coping, emphasizing how environmental factors and specific stressor characteristics influence individuals’ coping responses [12, 20, 27, 29].

Notably, home stressors were often perceived as uncontrollable or inescapable, leading to the use of less effective coping strategies such as *helplessness*, *avoidance-escape*, and *submission* [12, 27]. Similarly, in peer interactions, responses like *social isolation* and *opposition* were prevalent in dealing with peer aggression (Phelps, 2001, cited in [13]). In contrast, school stressors, perceived as more manageable, prompted the use of more constructive coping strategies, such as *problem solving* and *accommodation* [27]. Academic expectations and cultural norms, like respecting teachers, may have influenced participants to respond more positively to teacher-related stressors. However, *opposition*, characterized by behaviours like shouting and backbiting, was most commonly used in the school setting, possibly due to the perception of fewer negative consequences compared to other contexts.

Consistent with Zimmer-Gembeck and Skinner [30], participants most frequently used *problem solving* as a coping strategy across all settings, primarily through direct communication. This suggests that *problem solving* may be the most accessible strategy known to participants. While the high use of *problem solving* typically indicates better adjustment [15], there is a caveat, especially for adolescents living in poverty. Adaptive strategies like *problem solving* may not always be effective in all circumstances [27]. Some participants reported that *problem solving* and *support seeking* did not change their situation or led to unexpected outcomes, indicating a potential lack of experience or skills in using different coping strategies based on the situation or context. Accordingly, matching coping strategies to specific stressors may help participants adjust better (Folkman, 1984, cited in 31). In fact, Jaser et al. [31] found that problem solving and emotional expression are most adaptive for controllable stressors (e.g., peer conflicts), while acceptance or distraction are more adaptive for uncontrollable stressors (e.g., parental conflicts).

Our study found that participants across different age groups generally used similar coping strategies, with some minor differences, which is consistent with a previous study [28]. The 11–13-year-old participants showed a slightly higher use of *problem solving*, which aligns with typical developmental tendencies in early adolescence [30]. In contrast, the 14–16-year-old participants tended to use more maladaptive coping strategies, such as *helplessness* and *avoidance-escape*, and less instrumental coping, likely due to the emotional and cognitive vulnerabilities of middle adolescence. Older participants used more cognitive strategies like *accommodation* and *negotiation*, indicating a gradual improvement in coping strategy selection over the years [30]. They also showed a higher tendency to seek support when dealing with relationship stressors [13]. Additionally, while both genders showed a similar proportion of maladaptive and adaptive coping, girls tended to use more *avoidance-escape* and *helplessness*, while boys used more *opposition* and *social isolation*, consistent with Hampel and Petermann [32]. These differences may be attributed to the tendency of female adolescents to internalize conflict and male adolescents to externalize it through aggression [33].

Furthermore, it is important to interpret adolescents’ coping mechanisms within the Nepali sociocultural context. Nepal, a predominantly Hindu, collectivist society in South Asia, places great importance on values like filial piety, respect for elders, and obedience [34, 35]. In this society, expressing negative emotions, whether verbally or physically, is often discouraged from a young age [36]. Deviating from parental or teacher expectations is commonly seen as “misbehaviour,” leading to scolding, or even physical punishment [37, 38]. Adolescents in such a cultural setting may feel pressured to suppress their emotions and avoid conflicts with authority figures. As a result, when they encounter stress at home or school, many may have resorted to maladaptive coping strategies like *avoidance-escape*, *helplessness*, or *submission*, which manifest as withdrawn or silent behaviors [39]. Additionally, gendered socialization practices influence coping mechanisms, with different expectations for behavior based on gender: girls are often expected to be compliant and prosocial, while disruptive behavior may be more tolerated or expected from boys [38].

The results of this study could have significant implications for enhancing the psychosocial and mental well-being of adolescents in low-resource settings such as Nepal. First, our results highlight the need to promote more adaptive coping in adolescents experiencing or at risk of depression and anxiety. Adolescents often struggled to manage challenging stressors, especially those in the home environment that are beyond their control, such as inter-parental conflict, financial constraints, and parental abuse. Interventions should focus on helping adolescents develop healthier coping strategies to deal with these stressors and reduce reliance on maladaptive coping mechanisms. Teaching adaptive strategies can help adolescents replace maladaptive ones and improve their coping abilities (see [25]). For example, *accommodation* (adjusting preferences to fit environmental demands) is a more effective response to uncontrollable stressors than *submission* (yielding preferences to environmental constraints) [25]. By promoting adaptive coping mechanisms, we can support adolescents in navigating challenging situations and promoting their mental well-being.

Second, adolescents often struggle with effectively assessing stressors and choosing appropriate coping strategies. Research by Jaser et al. [31] emphasizes the importance of matching coping strategies to stressors to improve adjustment, prevent long-term mental health issues, and develop healthier coping skills. Merely teaching adaptive coping techniques is not enough. Despite the prevalence of maladaptive coping, adolescents typically attempt to select the best strategies for a given situation. Hence, interventions should concentrate on helping adolescents enhance their ability to evaluate stressors, assess the effectiveness of different coping strategies in their repertoire, and choose the most suitable and adaptive approach for each stressor.

Third, given the predominantly maladaptive nature of *desired* strategies and the frequent use of self-harming strategies (e.g., *submission*) and situational strategies (e.g., *helplessness*) in the home environment, there is a need for family-based interventions that focus on emotional regulation and conflict resolution for both adolescents and caregivers.

Finally, coping strategies and skills should also be incorporated into school curricula. However, since targeted education alone may not be sufficient (as adolescents often have knowledge about different coping strategies), school-based interventions should also include skills-based approaches to support them in developing practical and adaptive skills to manage challenges. The government of Nepal has introduced a school nurse program, which presents an opportunity to train school nurses in implementing interventions that focus on enhancing social skills and emotional development. 

Limitations of our study include the cross-sectional design, which may have hindered our ability to capture age-related trends fully. A longitudinal study would be better suited for this purpose. The study was conducted in a highly urban setting, so the generalizability of our findings may be limited in rural areas where the majority of adolescents in Nepal reside. The diversity in caste/ethnicity and cultural factors, especially concerning gender, could also restrict the generalizability of our findings. Additionally, the study only utilized self-reported data from adolescents regarding their experiences and coping strategies. There was no validation or cross-referencing of this information with observations or perspectives from caregivers. Incorporating caregiver insights could have enhanced the credibility and contextual interpretation of the results. Despite these limitations, our findings provide valuable insights that can be further investigated in future studies by refining interview questions to gather more comprehensive data.

## Conclusion

We have examined how adolescents in Nepal, living in urban poverty and at risk of depression and anxiety, navigate challenges in their home, school, and peer environments. The presence of *desired*, *actual*, and *ideal* coping strategies in adolescent narratives illustrates the dynamic nature of the coping process. Our study shows that these adolescents employed a combination of adaptive and maladaptive coping mechanisms, with slight variations based on age and gender. The adolescents predominantly relied on *problem* *solving* as a coping strategy, but also frequently resorted to *helplessness*, *avoidance-escape*, and *opposition*, indicating a lack of effective coping skills. Our findings underscore the importance of mental health interventions that focus on teaching adolescents adaptive coping strategies, stress appraisal, and the selection of appropriate coping mechanisms.

## Supplementary Information

Supplementary Material 1.

## Data Availability

Interested parties may notify the ALIVE (Improving Adolescent mentaL health by reducing the Impact of poVErty) investigators of their interest in collaboration, including access to the data-set analyzed here, through the following email: [emily.garman@uct.ac.za](mailto:emily.garman@uct.ac.za)

## Abbreviations

ALIVE: Improving Adolescent mentaL health by reducing the Impact of poverty
GAD-7: Generalized Anxiety Disorder
ICR: Inter-Coder Reliability
LMIC: Low- and Middle-Income Countries
PHQ-A: Patient Health Questionnaire adapted for adolescents
UNICEF: United Nations Children’s Fund

## References

[1] IHME. Global Burden of Disease Collaborative Network. Global burden of disease study 2021 (GBD 2021). Seattle. United States: Institute for Health Metrics and Evalutation (IHME); 2024.

[2] WHO. Mental health of adolescents: World Health Organization. 2024 [Available from: https://www.who.int/news-room/fact-sheets/detail/adolescent-mental-health

[3] United Nations DoEaSA, Population Division. World Population Prospects 2019: Highlights (ST/ESA/SER.A/423). 2019.

[4] Ridley M, Rao G, Schilbach F, Patel V. Poverty, depression, and anxiety: causal evidence and mechanisms. Science. 2020. 10.1126/science.aay0214. 33303583 10.1126/science.aay0214

[5] Marchi M, Alkema A, Xia C, Thio CHL, Chen LY, Schalkwijk W, et al. Investigating the impact of poverty on mental illness in the UK biobank using Mendelian randomization. Nat Hum Behav. 2024;8(9):1771–83. 38987359 10.1038/s41562-024-01919-3 PMC11420075

[6] World Bank. Poverty and shared prosperity 2022: correcting course. Washington, DC: World Bank; 2022.

[7] Shorey S, Ng ED, Wong CH. Global prevalence of depression and elevated depressive symptoms among adolescents: a systematic review and meta-analysis. Br J Clin Psychol. 2021;61(2):287–305. 34569066 10.1111/bjc.12333

[8] Yatham S, Sivathasan S, Yoon R, da Silva TL, Ravindran AV. Depression, anxiety, and post-traumatic stress disorder among youth in low and middle income countries: a review of prevalence and treatment interventions. Asian J Psychiatr. 2018;38:78–91. 29117922 10.1016/j.ajp.2017.10.029

[9] WHO. Suicide worldwide in 2019: global health estimates. World Health Organization; 2021.

[10] NHRC. National Mental Health Survey. Nepal-2020 (factsheets-adolescents) 2020 [Available from: https://nhrc.gov.np/publication/national-mental-health-survey-nepal-2020-factsheets-adolescents/

[11] Lazarus RS. FS. Stress, Appraisal, and coping. New York, NY: Springer; 1984.

[12] Cicognani E. Coping strategies with minor stressors in adolescence: relationships with social support, self-efficacy, and psychological well-being. J Appl Soc Psychol. 2011;41(3):559–78.

[13] Seiffge-Krenke I. Coping with relationship stressors: a decade review. J Res Adolesc. 2011;21(1):196–210.

[14] Skinner EA, Zimmer-Gembeck MJ. Ways and Families of Coping as Adaptive Processes. The Development of Coping2016. pp. 27–49.

[15] Evans GW, Kim P, Childhood Poverty C, Stress. Self-Regulation, and coping. Child Dev Perspect. 2012;7(1):43–8.

[16] Kim P, Neuendorf C, Bianco H, Evans GW. Exposure to childhood poverty and mental health symptomatology in adolescence: a role of coping strategies. Stress Health. 2016;32(5):494–502. 26234956 10.1002/smi.2646

[17] Santiago CD, Etter EM, Wadsworth ME, Raviv T. Predictors of responses to stress among families coping with poverty-related stress. Anxiety Stress Coping. 2012;25(3):239–58. 21614698 10.1080/10615806.2011.583347

[18] Lund C, Jordans MJD, Garman E, Araya R, Avendano M, Bauer A, et al. Strengthening self-regulation and reducing poverty to prevent adolescent depression and anxiety: Rationale, approach and methods of the ALIVE interdisciplinary research collaboration in Colombia, Nepal and South Africa. Epidemiol Psychiatric Sci. 2023;32(e69):1–8. 10.1017/S2045796023000811 PMC10803189 38088153

[19] Karkia B, Singh S. Formalizing the informal settlements in Kathmandu valley: a case of Bansighat along Bagmati river corridor. J Innov Eng Educ. 2022;5(1):64–76.

[20] Conover K, Daiute C. The process of self-regulation in adolescents: a narrative approach. J Adolesc. 2017;57:59–68. 28371653 10.1016/j.adolescence.2017.03.006

[21] Côté J, Salmela JH, Baria A, Russell SJ. Organizing and interpreting unstructured qualitative data. Sport Psychol. 1993;7(2):127–37.

[22] Skinner EA, Zimmer-Gembeck MJ. Ways and families of coping as adaptive processes. The development of coping. Cham: Springer; 2016. 10.1007/978-3-319-41740-0_2.

[23] Maxwell JA. Using numbers in qualitative research. Qual Inquiry. 2010;16(6):475–82.

[24] Sandelowski M. Real qualitative researchers do not count: the use of numbers in qualitative research. Res Nurs Health. 2001;24(3):230–40. 11526621 10.1002/nur.1025

[25] Skinner EA, Edge K, Altman J, Sherwood H. Searching for the structure of coping: a review and critique of category systems for classifying ways of coping. Psychol Bull. 2003;129(2):216–69. 12696840 10.1037/0033-2909.129.2.216

[26] Lazarus RS, Folkman S. Stress. Appraisal, and Coping. Springer; 1984.

[27] Zimmer-Gembeck MJ, Skinner EA. Adolescents’ coping with stress: development and diversity. Prev Researcher. 2008;15:3–7. 20344979

[28] Williams K, McGillicuddy-De Lisi A. Coping strategies in adolescents. J Appl Dev Psychol. 1999;20(4):537–49.

[29] Zimmer-Gembeck MJ, Skinner EA, Morris H, Thomas R. Anticipated coping with interpersonal stressors. J Early Adolesc. 2012;33(5):684–709.

[30] Zimmer-Gembeck MJ, Skinner EA. Review. The development of coping across childhood and adolescence: an integrative review and critique of research. Int J Behav Dev. 2010;35(1):1–17.

[31] Jaser SS, Champion JE, Reeslund KL, Keller G, Merchant MJ, Benson M, et al. Cross-situational coping with peer and family stressors in adolescent offspring of depressed parents. J Adolesc. 2007;30(6):917–32. 17241658 10.1016/j.adolescence.2006.11.010

[32] Hampel P, Petermann F. Age and gender effects on coping in children and adolescents. J Youth Adolesc. 2005;34(2):73–83.

[33] de Anda D, Bradley M, Collada C, Dunn L, Kubota J, Hollister V, et al. A study of stress, stressors, and coping strategies among middle school adolescents. Child Sch. 1997;19(2):87–98.

[34] An D, Eggum-Wilkens ND, Chae S, Hayford SR, Yabiku ST, Glick JE, et al. Adults’ conceptualisations of children’s social competence in Nepal and Malawi. Psychol Dev Soc. 2018;30(1):81–104. PMC6071425 30078957

[35] Burkey MD, Ghimire L, Adhikari RP, Wissow LS, Jordans MJ, Kohrt BA. The ecocultural context and child behavior problems: A qualitative analysis in rural Nepal. Social science & medicine (1982). 2016;159:73–82. 10.1016/j.socscimed.2016.04.020 PMC5201200 27173743

[36] Adhikari S, Rana H, Joshi MP, Cheng S, Castillo T, Huang KY. Parental wellbeing, parenting, and child mental health in families with young children in Arghakhanchi, Nepal. BMC Pediatr. 2025;25(1):6. 39762775 10.1186/s12887-024-05358-x PMC11702219

[37] Khadka J. Parental behavior across Nepali schools’ parents and children demographic characteristics. J Community Psychol. 2021;49(7):2704–18. 33450078 10.1002/jcop.22509

[38] Langer JA, Ramos JV, Ghimire L, Rai S, Kohrt BA, Burkey MD. Gender and child behavior problems in rural Nepal: differential expectations and responses. Sci Rep. 2019;9(1):7662. 31113970 10.1038/s41598-019-43972-3 PMC6529428

[39] Adhikari S, Ma J, Shakya S, Brøndbo PH, Handegård BH, Javo AC. Cross-informant ratings on emotional and behavioral problems in Nepali adolescents: a comparison of adolescents’ self-reports with parents’ and teachers’ reports. PLoS One. 2024;19(5):e0303673. 38753741 10.1371/journal.pone.0303673 PMC11098339

