# Supplementary material for: Coping strategies among poverty-affected adolescents experiencing or at risk of depression and anxiety in Nepal: a qualitative study
Source: BMC Psychol. 2025 Dec 1;13:1325. doi: 10.1186/s40359-025-03698-6 (PMC12670861; doi:10.1186/s40359-025-03698-6)
Supplement: Supplementary file 1 — Supplementary Material 1. [file 40359_2025_3698_MOESM1_ESM.docx]

**SEMI-STRUCTURED INTERVIEW GUIDE**

| **THEMES** | **KEY QUESTIONS** | **BREAKDOWN OF KEY QUESTIONS** | **PROBING QUESTIONS** |
| --- | --- | --- | --- |
| **Scenario 1: At School: "My teacher made me angry”**  ***(This reaction is shown by your friend of similar age and class.)*** | | | |
| Reason to lead that reaction | What you think might have happened to lead to that reaction?  Why these problems might have happened at school for (friend)? | What might they have said or done?   What do you think he/she was feeling (heart-mind perspective)?  What do you think he/she was thinking (brain-mind perspective)?  What do you think happened before the *[*issue*]* that caused it? | *Probe to make sure they explain the story, and what emotions his/her friend felt? Examples of Probes: -reasons behind his/her anger?  -teacher done or said that made (friend) angry? (behavior/nature/activities)  -(friend)'s behaviour, academic performance, classroom activities, gossiping, lack of discipline, extracurricular activities, out of school activities (gang fight, teasing, bullying), financial dues/problems, , favoritism & bias by teacher*  *- household problems of students* (*Probe if they struggle to answer, for example was it problems with their teachers/friends/parents?)* |
| Advise to friend | How would you advise your friend to deal with the situation? | What would you tell them to do?  Why would you tell them to do this?  What would happen if they followed your advice? | *Probes examples: - your suggestions  - Impact on his/her heart-mind (man), brain-mind (dimaag) after your suggestions* |
| Self-experience of similar situation | Have you seen or experienced something like this in your life?  How did you react?  *(If no similar experiences, probe for placing him/her in the situation and ask how would they react.)* | How did you feel? What did you do in that situation?  How did that situation affect you?  Did you share your problem with anybody? | *Probe to make sure he/she explains the story, and what emotions he/she felt?*  ***If (participant) has similar experiences***   *- Probes similar as above (used for (friend))*  *- Sharing the problems/feelings (best friend, father, mother, siblings, relatives, classmates, principal, directly to the teachers, on social media).*  *- Suggestions you received (followed or not /helpful or not)*  *If the participant had not shared with anyone, probe for whom he/she would share.* |
| Comparing the responses when it was you in this situation and when it was your friend | Do you notice any differences in how you would/had react/reacted, and how you would/had advise/advised your friend to react?  Why the responses for the same situation differed for you and your friend?  *(*If they are the same, ask them to elaborate why they are the same*)* | What are the differences between these two strategies/responses?  What do you think about these differences?  Why do you think they are different?  Why would/had not you give/given similar advices to your friends? | *If needed, explain: Remember you said that you would do [xxx] if it was you, but you said that you would advise your friend to do [xxx]. Have I got that right? Did I miss anything out?*  *Probes: interviewer notes some differences in the responses, asks them to explore these differences, the reasons behind the differences* |
| **Scenario 2: At home: “My Mum is making me really. Upset”**  **(Initially asking about current members in the household and using appropriate caretaker (Mum/Father/etc.) for the above scenario.)** | | | |
| Reason to lead that reaction | What you think might have happened to lead to that reaction?  Why these problems might have happened at home for (friend)? | What might they have said or done?   What do you think he/she was feeling (heart-mind perspective)?  What do you think he/she was thinking (brain-mind perspective)?  What do you think happened before the *[*issue*]* that caused it? | *Probe to make sure they explain the story, and what emotions his/her friend felt? Examples of Probes: - reasons behind his/her upset?  - mum done or said that made (friend) upset? (behavior/nature/activities of mum)  -problems that led to disturbed relationship between mum and children Eg: family conflict, substance/alcohol abuse, financial problem, pending fees, gender discrimination, extra-marital affairs, restrictions from parents/mum, unfulfilled desire. -Friend's behaviour, academic performance, out of school activities (gang fight, teasing, bullying), teenage intimate relationship. Probe if they struggle to answer, for example was it problems with their mum/friends/other family members?* |
| Advise to friend | How would you advise your friend to deal with the situation? | What would you tell them to do?  Why would you tell them to do this?  What would happen if they followed your advice? | *Probes examples: - your suggestions  - Impact on his/her heart-mind (man), brain-mind (dimaag) after your suggestions* |
| Self-experience of similar situation | Have you seen or experienced something like this in your life?  How did you react?  *(If no similar experiences, probe for placing him/her in the situation and ask how would they react.)* | How did you feel? What did you do in that situation?  How did that situation affect you?  Did you share your problem with anybody? | *Probe to make sure he/she explains the story, and what emotions he/she felt?*  ***If (participant) has similar experiences***   *- Probes similar as above (used for (name)*  *- Sharing the problems/feelings with (best friend, father, mother, siblings, relatives, classmates, on social media).*  *- Suggestions you received (followed or not /helpful or not)*  *If the participant had not shared with anyone, probe for whom he/she would share.* |
| Comparing the responses when it was you in this situation and when it was your friend | Do you notice any differences in how you would/had react/reacted, and how you would/had advise/advised your friend to react?  Why the responses for the same situation differed for you and your friend?  *(*If they are the same, ask them to elaborate why they are the same*)* | What are the differences between these two strategies/responses?  What do you think about these differences?  Why do you think they are different?  Why would/had not you give/given similar advices to your friends? | *If needed, explain: Remember you said that you would do [xxx] if it was you, but you said that you would advise your friend to do [xxx]. Have I got that right? Did I miss anything out?* *Probes: interviewer notes some differences in the responses, asks them to explore these differences, the reasons behind the differences* |
| **Scenario 3: With friends: “My friend hurt my feelings”** | | | |
| Reason to lead that reaction | What you think might have happened to lead to that reaction?  Why these problems might have happened at school for (friend)? | What might they have said or done?   What do you think he/she was feeling (heart-mind perspective)?  What do you think he/she was thinking (brain-mind perspective)?  What do you think happened before the *[*issue*]* that caused it? | *Probe to make sure they explain the story, and what emotions his/her friend felt?*  *Examples of Probes: - reasons behind his/her pain (hurt)?  - friend done or said that made (name) hurt? (behavior/nature/activities of friend)  - problems that led to disturbed relationship between two friends*  *-(name)'s behaviour, academic performance, classroom activities, out of school activities (gang fight, teasing, bullying)*  *Probe if they struggle to answer, for example was it problems with their friends/relatives/parents/teachers?* |
| Advise to friend | How would you advise your friend to deal with the situation? | What would you tell them to do?  Why would you tell them to do this?  What would happen if they followed your advice? | *Probes examples: -your suggestions  - Impact on his/her heart-mind (man), brain-mind (dimaag) after your suggestions* |
| Self-experience of similar situation | Have you seen or experienced something like this in your life?  How did you react?  *(If no similar experiences, probe for placing him/her in the situation and ask how would they react.)* | How did you feel? What did you do in that situation?  How did that situation affect you?  Did you share your problem with anybody? | *Probe to make sure he/she explains the story, and what emotions he/she felt?*  ***If (participant) has similar experiences***   *- Probes similar as above (used for (friend)*  *- Sharing the problems/feelings (father, mother, siblings, relatives, classmates, principal, directly to the teachers, on social media).*  *- Suggestions you received (followed or not /helpful or not)*  ***-*** *feeling when your friend left you while going out to travel/broke your things/gave more time to others than you? If the participants had not shared with anyone, let them to imagine again, and probe as above  If the participant had not shared with anyone, probe for whom he/she would share.* |
| Comparing the responses when it was you in this situation and when it was your friend | Do you notice any differences in how you would/had react/reacted, and how you would/had advise/advised your friend to react?  Why the responses for the same situation differed for you and your friend?  *(*If they are the same, ask them to elaborate why they are the same*)* | What are the differences between these two strategies/responses?  What do you think about these differences?  Why do you think they are different?  Why would/had not you give/given similar advices to your friends? | *If needed, explain: Remember you said that you would do [xxx] if it was you, but you said that you would advise your friend to do [xxx]. Have I got that right? Did I miss anything out?   Probes: interviewer notes some differences in the responses, asks them to explore these differences, the reasons behind the differences.* |

**Diary Writing Activity**

We just recently interviewed you. During that interview, we discussed the problems or difficulties that you have experienced at school, at home or with friends. We also talked about the feelings you had, the reactions you showed and your behavior in the situation, and the advice you gave to your friends to deal with the situation.

In the coming days, you may again experience similar problems/difficulties in your daily life we discussed before. In order to know and understand the problems or difficult situations that made you upset or anxious or angry, we will let you write a diary. In your diary, you can write about your problems or difficult situations, how you felt when you experienced those problems, how you reacted in the situation and how you behaved in that situation. The information you provide will help us to develop programs designed to prevent mental health problems such as depression and anxiety in adolescents (10-19 age group).

We will come every week to collect the information you have written in the diary. In that time, you can ask us questions about anything you haven’t understood or felt difficulty about, or any queries you may have. We will try to explain in detail anything that you did not understand. All information you provide will be kept confidential and will not be shared with your friends and/or family. The information will only be used by the researchers.

We urge you to write about your experience or thoughts without any hesitation on some of the questions provided below. These questions are similar to the ones we asked you during the interview. Nothing written in this diary will be considered right or wrong. We simply want to know about your experiences and thoughts, so feel free to write about them without hesitation.

**Diary Writing**

| 1. Did you experience any problems/difficulties or challenges this week at home, at school, or among friends that made you feel bad, sad, frustrated, or angry?   (If you did not experience any such problems, please state that in the diary. If you did, please explain the problem in detail in the diary.) |
| --- |
| 1. When you experienced that problem/difficult situation, what did you do? Did your reaction/response help you to deal with the situation? If it did, could you please explain how did it help? If it didn’t, why do you think it didn’t help? |
| - 1. How were you feeling in that situation?   2. What type of thoughts were you having? |
| 1. If your friends/siblings were in a similar situation as you. What advice would you give them? / What would you tell them to do? |
| 1. Have you seen any problems among your friends/siblings at home, school, or among friends this week? can you explain what happened in detail? What advice did you give them / What did you tell them to do? |
